# Investigating Cutaneous Squamous Cell Carcinoma *in vitro* and *in vivo*: Novel 3D Tools and Animal Models

**DOI:** 10.3389/fmed.2022.875517

**Published:** 2022-05-09

**Authors:** Marika Quadri, Alessandra Marconi, Simran K. Sandhu, Alexi Kiss, Tatiana Efimova, Elisabetta Palazzo

**Affiliations:** ^1^DermoLAB, Department of Surgical, Medical, Dental and Morphological Science, University of Modena and Reggio Emilia, Modena, Italy; ^2^Department of Anatomy and Cell Biology, George Washington University School of Medicine and Health Sciences, Washington, DC, United States; ^3^The George Washington Cancer Center, George Washington University School of Medicine and Health Sciences, Washington, DC, United States; ^4^Department of Dermatology, George Washington University School of Medicine and Health Sciences, Washington, DC, United States

**Keywords:** cSCC *in vitro* modeling, spheroids, 3D models, zebrafish, carcinogenesis mouse models

## Abstract

Cutaneous Squamous Cell Carcinoma (cSCC) represents the second most common type of skin cancer, which incidence is continuously increasing worldwide. Given its high frequency, cSCC represents a major public health problem. Therefore, to provide the best patients’ care, it is necessary having a detailed understanding of the molecular processes underlying cSCC development, progression, and invasion. Extensive efforts have been made in developing new models allowing to study the molecular pathogenesis of solid tumors, including cSCC tumors. Traditionally, *in vitro* studies were performed with cells grown in a two-dimensional context, which, however, does not represent the complexity of tumor *in vivo*. In the recent years, new *in vitro* models have been developed aiming to mimic the three-dimensionality (3D) of the tumor, allowing the evaluation of tumor cell-cell and tumor-microenvironment interaction in an *in vivo*-like setting. These models include spheroids, organotypic cultures, skin reconstructs and organoids. Although 3D models demonstrate high potential to enhance the overall knowledge in cancer research, they lack systemic components which may be solved only by using animal models. Zebrafish is emerging as an alternative xenotransplant model in cancer research, offering a high-throughput approach for drug screening and real-time *in vivo* imaging to study cell invasion. Moreover, several categories of mouse models were developed for pre-clinical purpose, including xeno- and syngeneic transplantation models, autochthonous models of chemically or UV-induced skin squamous carcinogenesis, and genetically engineered mouse models (GEMMs) of cSCC. These models have been instrumental in examining the molecular mechanisms of cSCC and drug response in an *in vivo* setting. The present review proposes an overview of *in vitro*, particularly 3D, and *in vivo* models and their application in cutaneous SCC research.

## Introduction

Cutaneous Squamous Cell Carcinoma (cSCC) represents the second most frequent type of non-melanoma skin cancer (NMSC), after basal cell carcinoma (BCC) (1–4). cSCC incidence is constantly increasing worldwide, up to 200 percent in the past 30 years (5). An estimated number close to 2 million cases are diagnosed every year only in the United States, which means about 205 cases are diagnosed every hour (6). cSCC may develop either on healthy tissues, or on a pre-existing actinic keratosis (AK) or on a burn scar. Notably, the number of people with AK is increasing rapidly and the AK affects more than 60% of the elderly population. Therefore, the increase in average life expectancy in the era of industrialization means that the cSCC represents a major public health problem, with a diagnosis that remains differential and requires biopsy.

Cutaneous squamous cell carcinoma presents a spectrum of different histopathological stages, going from *in situ* to invasive and metastatic diseases. While surgical resection is usually curative for the less aggressive tumors, such as the *in situ* cSCC, the treatment of the advanced lesions require multi-modal treatment consisting of surgery, radiation, chemotherapy, and/or targeted or immunotherapy (7, 8). As the resection of the advanced lesions might be significantly disfiguring for the patients, psychological treatments to counteract stress-dependent effects on tumor progression might also be beneficial (9). Alarmingly, this is accompanied by a sustained decline of the disease-free survival rate (7). In addition to the surgical approach, other techniques, such as curettage, cryosurgery, or photodynamic therapy (PDT) are also used for the non-invasive cSCC forms. In the case of the advanced disease, when surgery is not a good option, radiotherapy becomes also indicated (8). In the recent years, due to use of novel technologies, the study of the mechanisms of cSCC development and progression is becoming more informative and accurate. For example, the association between dermoscopy and reflectance confocal microscopy facilitates the diagnosis (10–14) and, therefore, accelerates the beginning of the treatment.

An altered balance between keratinocyte proliferation and differentiation leads to the perturbation of the epidermal homeostasis, inflammation and epidermal hyperplasia, which can promote cSCC development over time, followed by intradermal dissemination of the malignant cells (15, 16). By combining single cell sequencing technologies and CRISPR screening of the tumor specific gene networks in human cSCC versus normal healthy skin, a cluster of tumor specific keratinocytes has been identified which are unable to fully undergo differentiation, proliferate more rapidly at the basal layer and express epithelial to mesenchymal transition (EMT) associated genes (17).

The pathogenesis of cSCC is likely to involve a combination of environmental and molecular factors (18). The Ultraviolet radiation (UVR) from sun exposure is considered the main environmental risk factor, and the UV-induced *TP53* mutations are early events in cSCC development and are responsible for a major genomic instability (19–21). Other common genetic changes involved in cSCC initiation are mutations in *CDKN2A*, *NOTCH* and *RAS* as well as several other genes associated with cell cycle regulation (*CDKN2B*), signaling pathways that control proliferation, survival (*PIK3CA, PTEN, EGFR*), and squamous differentiation (*TP63, SOX2*), and epigenetic regulators (*KMT2C, KMT2D*) (16, 22–28). Immunosuppression, chronic wounds, and BRAF inhibitors for the treatment of melanoma are among additional risk factors for cSCC (28).

Given the complexity of the cSCC tumors, both *in vitro* and *in vivo* models are required to obtain a detailed understanding of the molecular processes leading to cSCC development, progression, and invasion. This review will focus on the description of the most important tools for the study of cSCC, starting from the bi-dimensional classic 2D culture system to the 3D culture, such as spheroids, organoids and organotypic cultures. Although *in vitro* models demonstrate high potential to enhance the overall knowledge in cancer research, they lack systemic components, which may be solved only by using animal models. In particular, the zebrafish xenografting model is emerging as an alternative approach in cancer research, offering high-throughput drug screening and live cell invasion imaging. Furthermore, over time several types of mouse models, genetically modified or chemically treated, were developed for both basic and translational research, representing an invaluable resource for cSCC therapy development and testing.

## Oral Versus Cutaneous Squamous Cell Carcinoma

The development of *in vitro* and *in vivo* tools by using cutaneous rather than oral cancer-derived primary cells is determinant for explorative and functional studies within cSCC, and in this context, oral SCC (oSCC) cells have been independently used. “Squamous cancer” is usually referred to a heterogeneous group of malignancies, including those arising from epidermal keratinocytes of the skin, mucosal tissues of the head and neck region (such as tongue or pharynx), lung or esophagus. Specifically, SCC from the oral cavity includes neoplasms of the base of the tongue and of the hard and soft palate, which are also part of the oropharynx.

**FIGURE 1:**
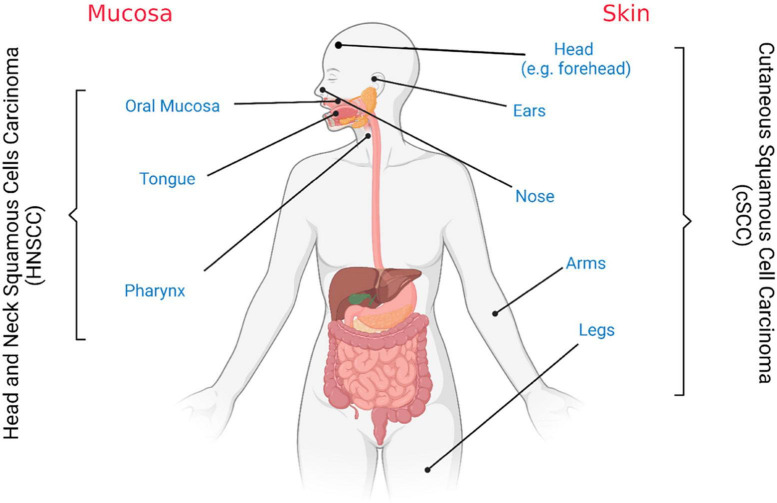
Cutaneous and oral squamous cell carcinoma tissues of origin. Schematic representation. Figure created with BioRender.com.

Therefore, the use of the word “cutaneous” allows the identification of the origin of such pathology, which reflects a different diagnostic and therapeutic path (Figure 1). For example, it is well-established that cSCC represents a major health issue among people with fair skin, mostly male and immunocompromised and with a 5% of lymph node metastasis (29); on the other hand, oSCC, arising from the oral cavity, is usually associated with a higher rate of nodal metastasis (10–65%) (30, 31). Moreover, even if the overall survival (OS) rates associated with the metastatic diseases are similar for cutaneous and oral SCC, patient comorbidities determine the disease specific survival (32).

By looking at the intrinsic characteristics of the tissue, skin and mucosa present substantial variations in terms of thickness and level of keratinization, as well as vasculature distribution (33). E.g., the hard palate and gingiva are partially keratinized epithelia and the tongue is covered by a specialized epithelium that includes the taste buds. Overall, epidermis and mucosa undergo to tissue-specific mechanical stress and exposure to environmental stimuli, including contacts with secretes (such as mucus) and enzymes characteristic of the tissue, as well as pathogens. Furthermore, with regard to keratin expression in neoplastic versus healthy tissues, both proteomic and transcriptomic profiles reveal the presence of a tumor dependent signatures, with a defined keratin pattern associated with proliferation and differentiation status of the cells (34–36). For example, while Keratin 13 is absent in cancers from the gingivo-buccal complex (35), it becomes indicative of a neoplastic and malignant conversion in skin cancers (37–39).

## *In vitro* Models

**FIGURE 2:**
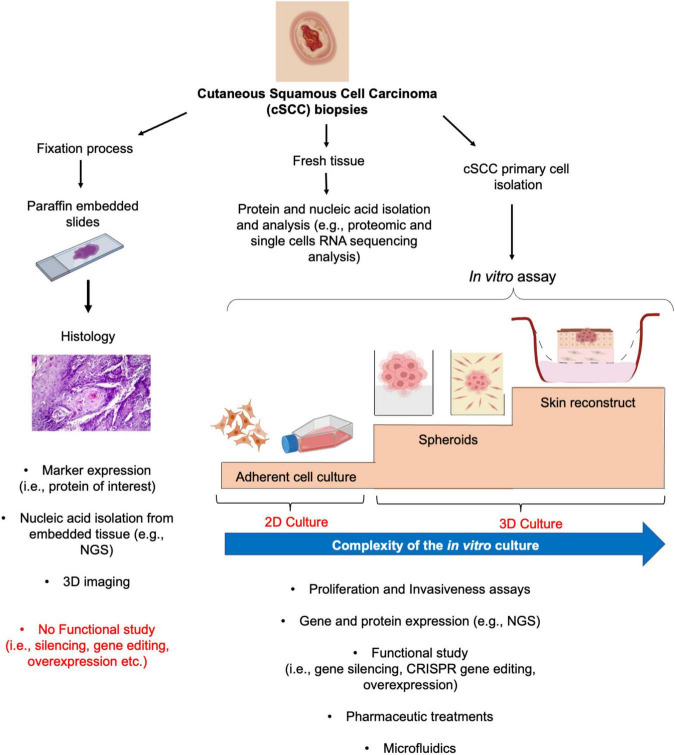
From histology to 2D and 3D culture methods for cSCC study. From fresh biopsy, histological evaluation provides the basis for subsequent gene and protein expression analysis **(left panel)**. Similarly, fresh tissue could undergo to different procedures for protein or gene expression analysis **(middle panel)**. The isolation and culture of the cSCC primary cells allows the functional characterization of the tumor by mean of 2D and more sophisticated 3D culture systems, such as spheroids and skin reconstruct **(right panel)**.

Tumors are highly complex diseases that require a deep understanding of the underlying mechanisms to ultimately provide the best possible care for patients. Therefore, the identification of early biomarkers and molecular drivers of the cSCC advanced conditions demand for several pre-clinical studies to discover and characterize novel and potential targets of therapies. In the recent years, intensive efforts have been made for the development of new tools for cancer research (40, 41). In the present review, we discuss several models with different order of complexity to study and manage cSCC (Figure 2).

As common in all hospital routine procedure, when a cSCC lesion is surgical removed, it can be fixed and embedded in paraffin for diagnoses (42). Typically, tumoral fixed section are used for the evaluation of the histology of the lesion and of well-defined marker expressions, through immunohistochemistry or immunofluorescence by diagnostic laboratories (Figure 2, left panel). From the oncological research point of view, this analysis defines the clinical aspect of the tumor that could be subsequently correlated with the expression of novel targets, allowing their validation within an *ex vivo* neoplastic setting. The screening of a high number of AK, *in situ* and well to poorly differentiated cSCC has demonstrated the localization and expression of molecules associated with cell cycle, differentiation and inflammatory status of the tumors (39, 43–45). For example, the nuclear localization of the p53 downstream target survivin correlates with poorly differentiated and more invasive cSCC as compared to well-differentiated cSCC and AK (43). Taking advantage of these findings, subsequent studies have shown a role for survivin in cSCC cancer stem-like cells (46). Moreover, thanks to the relatively recent development of protocols for nucleic acid extraction from embedded tissues, it is also possible to isolate specifically localized areas, starting from the histological evaluation, and to dissect their molecular profile at the transcriptomic level by next generation sequencing (NGS) technologies. These methods allow large scale comparisons between samples presenting defined features, thus promoting the identification, and further characterization, of the differentially expressed genes (47). Tumoral tissues could be directly analyzed for protein and gene expression by dedicated protocols (Figure 2, middle panel). A further evolution of the NGS techniques, for example, such as single-cell RNA sequencing (sc-RNAseq) technology combined or not with spatial transcriptomic, allows to decipher the molecular pattern of the cSCC cells (17, 48). Recently, by gene set enrichment analysis from sc-RNAseq, it has been shown that cSCC cells and keratinocytes possess significantly distinct gene expression patterns and chromosomal copy number variation. Moreover, 18 hallmark pathways of cancer have been shown to be upregulated in cSCC compared to normal keratinocytes (48).

On the other hand, those evaluations do not permit a direct functional study. For this reason, through the use of adherent (2D) and 3D culture systems, the roles of molecules of interest in control of proliferation, progression, and invasion of cSCC cells could be clarified (Figure 2, right panel). The isolation of human cSCC cells has been initially described by Rheinwald and Beckett in 1981, with the isolation and establishment of the SCC12 and SCC13 cell line as coculture on feeder layer of 3T3 NIH fibroblasts (49). Starting with this method, different laboratories had further optimized a protocol for primary cSCC cell isolation and culture (50–53) and different cSCC cell lines have been established and used for cSCC research (Table 1).

**TABLE 1:** Human and murine cSCC cell lines.

Name	Origin	Features and research	References
A-431	Epidermal carcinoma of the vulva from 85-years-old female	Epidermoid. adherent, hypertriploid cells Adhesion-related molecule studies Anti-proliferative effect of drugs	Giard et al. (65) Pfaff et al. (105); Liu et al. (67) Qiao et al. (234), Kang et al. (235)
DJM-1	Malignant trichilemmal cyst cells from 87-years-old female	Epidermal carcinoma, adherent, cell Adhesion and cell spreading Molecular profile (gene or protein expression)	Kitajima et al. (69) Wakita et al. (236); Mizutani et al. (71) Egashira et al. (72); Yanagi et al. (73)
SCC12	Cutaneous SCC from 60-year-old male	Epidermal carcinoma, adherent, cell Analysis of proliferation/invasion Molecular profile *In vivo* studies	Rheinwald and Beckett, (49) Zhang et al. (237) Li et al. (238) Shin et al. (239)
SCC13	Cutaneous SCC from 56-year-old male	Epidermal carcinoma, adherent, cell Analysis of proliferation/invasion Pharmacological studies *In vivo* studies	Rheinwald and Beckett, (49) Commandeur et al. (144) Hah et al. (74) Zhang et al. (237) Dallaglio et al. (43) Lotti et al. (46)
HSC-1 HSC-5	Cutaneous SCC from the hand of a 75-years-old male	Epithelial-like cell morphology, maintained *in vitro* in continuous tissue culture for over 2 years cSCC related functional studies Analysis of proliferation/invasion Pharmacological studies	Hozumi et al. (76) Kondo and Aso, (240) Hu et al. (80) Kanzaki et al. (79) Mikami et al. (78) Deng et al. (241)
MET1, MET2, MET3, MET4	Cutaneous acantholytic squamous cell carcinoma from the back of the left hand (first excision—MET1) Recurrent cSCC (second excision MET2; third excision—MET3) Metastatic cSCC within left axillary lymph nodes that were biopsied following the third excision (MET4)	Keratinocyte cell morphology, adherent cells Genetic analysis of the malignant process Tumor progression studies Analysis of proliferation/invasion Pharmacological studies	Proby et al. (81) Green et al. (242) Verschooten et al. (243) Kopecki et al. (244) Chen et al. (245)
IC1	Moderately differentiated primary cSCC from the right temple of 77-years-old male immunocompetent patient	Keratinocyte cell morphology, adherent cells Genetic analysis of the malignant process Tumor progression studies Analysis of proliferation/invasion Pharmacological studies	Inman et al. (82) McHugh et al. (246) South et al. (22) Watt et al. (50) Wang et al. (247)
T11	Poorly differentiated cSCC from 48-years-old male transplanted patient	Keratinocyte cell morphology, adherent cells Genetic analysis of the malignant process Tumor progression studies	Inman et al. (82) McHugh et al. (246)
XL50	UVR-induced cSCC in SKH-1 hairless mice	Keratinocyte cell morphology, adherent cells Functional studies related to IV dependent development of SCC and response to therapy	Das et al. (248) Shi et al. (90) Zhang et al. (89)

The use of primary cSCC cells should be done in the early passages to avoid a progressive adaptation to monolayer culture conditions and consequent modification of the cells in terms of growth or response to stimuli. Progress in cancer research provide evidence that cancer cells, including cSCC cells (52), can grow as spheroids or organoids (54–58), indicating that it is possible to maintain the architecture of cell-to-cell interaction as well the tissue organization with respect to the external environment, which is important for both functional studies, based on gene silencing or overexpression or gene editing, and drug development (59). The use of spheroids also permits the evaluation of cancer cell invasiveness through cellular or acellular matrix-based assays (60–62). Therefore, this system can guarantee a more homogeneous cellular behavior as compared to the bi-dimensional scratching assays, which mimic a more wound-like response. The complexity of the cSCC and the interaction of the cSCC cells with the dermal compartment could be, in the end, more faithfully reproduced using the skin reconstruct (63, 64). These advanced 3D models can be, in fact, engineered according to the experimental requirements and provide a direct analysis of the greater or lesser proliferative or aggressive phenotype of the cSCC cells.

### Bi-Dimensional Cutaneous Squamous Cell Carcinoma Cell Cultures

The use of clinical samples for cSCC cell isolation involves, of course, both ethical and practical problems. Therefore, the establishment of tumor cell lines is always a good option for the optimization of culture protocols and drug testing, before using primary cells from patients.

The pioneering work of Giard and collaborators (65), back in 1973, allow the establishment of 13 tumor cell lines, starting from more than 200 different tumor types, including carcinomas, sarcoma, melanoma, and brain neoplasms. From this work, the epidermoid carcinoma cell line A-431, derived from an 85-year-old female, started, and kept being used for experiments related to cSCC (66–68). Similarly, the epithelial cell line DJM-1, specifically derived from a malignant trichilemmal cyst cell, from an 87-years-old Japanese female (69), is considered as a good model for cSCC (70–73).

In 1981, Rheinwald and Beckett described the isolation and characterization of six human SCC of the epidermis and the tongue, by using clonal growth and serial cultures (49). Thanks to these experiments, SCC12 and SCC13 cell lines, which were derived from surgically removed facial skin SCC lesions from a 60-year old male kidney *trans* plant recipient, treated with immunosuppressive drugs for the previous 7 years, and a 56-year-old female who had received a series of radiation treatments for several years before the surgical removal, respectively, were subsequently derived and widely used for cSCC research since (43, 46, 74, 75).

In the same years, the cell lines HSC-1 and HSC-5 have been derived from a 75-years-old male and maintained *in vitro* in continuous tissue culture for over 2 years (76, 77). HSC-5 cell line has been recently used to evaluate the role of TRL-4 (Toll-like receptor 4) in cSCC cells as regulator of the immune response and its correlation with malignancy level of cancer cells (78). HSC-1 and HSC-5 cells have also been used to look at the expression of IGF2BP3 (Insulin Like Growth Factor 2 mRNA Binding Protein 3) as a biomarker to distinguish between cSCC and keratoacanthoma (79) or to evaluate the role of CXCR7 expression in cSCC (80).

More recently, a distinct series of cSCC cell lines, named from MET1 to MET4, has been obtained by Leigh lab from 4 different squamous cell carcinomas, representing a primary tumor, two local recurrences and a distant metastasis of invasive SCC (81). It has been shown these MET cell lines required reduced growth supplements and presented altered differentiation, with anormal keratin 18 expression, which is consistent with the keratin expression profile of the original tumor. Having such cell lines from the same patient allow the study of tumor progression and help in the identification of the molecular events leading to cSCC development. Together with the previous, IC1, a cSCC cell line from a 77-years-old male immunocompetent patient and the T11 cSCC cell line, derived from a 48-years-old male transplanted patient has been used for path discovery and drug susceptibility studies (82, 83).

This initial overview highlights the fact that there is a strong interest in obtaining cSCC cell lines for pre-clinical research. In fact, those cSCC patients that develop advanced disease, with metastasis, are treated with a combination of surgery and radiation therapy, but there are few successful therapies for the refractory tumors. Therefore, several laboratories work extensively for the establishment of both primary and metastatic cSCC cell lines (83, 84). Furthermore, the use of the skin derived SCC cells assumes a significant meaning also in the context of those SCCs arising from a genetic condition, such as the RDEB (recessive dystrophic epidermolysis bullosa) where a targeted therapy approach is required (85).

Moreover, the development of the HaCaT cell line (86), a spontaneously immortalized human epithelial cell line from adult skin, which remains non-tumorigenic and maintains differentiation capacity, provided a useful model to study the mechanisms of neoplastic transformation *in vitro* (63, 87).

As for murine cSCC-derived cells, there are a lower number of models as compared to the humans given the opportunity of developing several mouse models of cutaneous carcinogenesis *in vivo*, as will be discussed in the next sections of the present review. In 1994, Ruggeri and coworkers described the analysis of six murine cSCC cell lines (BPCC), obtained following a benzo[a]pyrene mediated carcinogenesis protocol (88). Molecular and functional analysis of these cells indicated that they could be used for the evaluation of the preneoplastic conditions (88). A note should be given to the XL50 cSCC cell line, established from UVR-induced cSCC in SKH-1hairless mice, that have been used to demonstrate the role of retinoic acid receptor-related receptor alpha (Rorα) in promoting the progression from AK to UVR-induced cSCC (89). Moreover, they have been implied in experiments related to testing the efficacy and safety of the X-PDT therapy (90).

A summary of the main features and their prevalent use for the human and mouse cSCC cell lines above described are reported in Table 1.

As summarized in Table 1, cSCC cultures notably contributed to the discovery of several molecular targets with the subsequent drug development. For example, it is known that in cSCC tumors, Epidermal Growth Factor Receptor (EGFR) is strongly involved in the maintenance of the hyperproliferative condition and its overexpression, and amplification or overactivation in SCC cells as compared to normal keratinocytes have been reported (25). Similarly, the expression of the insulin-like growth factor-I receptor (IGF-IR) has been associated with the higher grade cSCC (91). Treatment with EGFR inhibitor, such as cetuximab, combined with the anti-IGF-IR antibody, has been shown to reduce proliferation and induced apoptosis in cSCC cells *in vitro*, showing that this approach could be useful for the treatment of cSCC (92).

### Three-Dimensional Models: Existing Methods and Applications to Cutaneous Squamous Cell Carcinoma

Although cSCC 2D adherent monolayer culture are still widely used, the use of 3D models is gradually emerging. Solid tumors growth in a 3D conformation, where cell-cell and cell-matrix interaction are present, and cells are exposed to vary biochemical gradients. In adherent monolayer condition, cells are homogenously subjected to environmental changes or molecules (e.g., oxygen, growth factors, nutrients), while 3D cultures better recapitulate the situation *in vivo* mimicking their three-dimensional architecture (41, 59). Additionally, solid tumors are circumscribed by surrounding cellular (e.g., stromal, immune, connective, and vascular cells) and non-cellular component (e.g., extracellular matrix—ECM, growth factors, cytokine etc.), which forms the tumor microenvironment (TME). It is widely known that TME plays a crucial role in tumor development, progression, and metastasis (93). Therefore, it is clear the necessity of considering TME in cancer studies.

Over the recent years, spheroids, organoids, and organotypic 3D cultures have been developed, contributing to the comprehension of the molecular mechanisms underlying neoplastic diseases, including cSCC. In addition, these models significantly contribute to novel drug testing and pharmaceutical development, due to their ability to more accurately mimic the human disease pathology and more accurately respond to treatments.

#### Spheroids and Organoids

Spheroids represents the basic method in 3D culturing. Here, tumoral cells (cell lines or primary cells) are fortified to interact within each other, thus recapitulating the *in vivo* milieu of tumor cell-cell interactions, growth features and metabolic gradients (40, 41, 94). In recent years, given the highly attractive potential for cancer research, several approaches have been developed to generate spheroids (40, 41, 95, 96). In general, these techniques could imply both that tumoral cells are cultured avoiding the adherences to the plastic surface (scaffold-free methods) or are grown on or within matrices (scaffold-based methods). The most common scaffold-free method is the liquid overlay method, where cells are seeded on a culture plate coated with an inert substrate (e.g., agarose or polyHEMA). In these conditions, tumoral cells spontaneously aggregate to form a spherical 3D structure. Alternatively, the hanging drop cell culture method can be used, which involves a deposition of a drop of cell suspension (20–40 μl) on an inverted lid, where cells, due to the surface tension and gravitational forces, spontaneously aggregate forming spheroid at the bottom of the drop (97). However, not all tumoral cells are able to forms spheroids using these methods. Some of them may require specific matrices or scaffolds, such as collagen, matrigel or synthetic scaffold (e.g., polyethylene glycol - PEG-, polylactic-co-glycol -PLGA, etc.) (96, 98).

Recently, tumor derived spheroids have been prepared from different cancer cell types (99–102), including cSCC (52, 103), and have been widely employed for the study of tumor pathology, progression, invasion, and for drug screening and development (60, 61, 98, 104).

Pfaff and co-workers showed that T-cadherin silencing increases motility and invasive potential in cSCC spheroids, which was completely reverted by its overexpression (105). In several studies, a spheroids formation assay was established to evaluate the stem cell-like function of cSCC cells *in vitro* (53, 106). Silijamaki and co-workers developed a co-cultured spheroid model comprising of primary skin fibroblasts and HaCaT cells transduced with H-Ras. They showed that fibroblast increases the invasive capacity of transformed HaCaT cells in collegen I matrix *via* Ras/TGFβ signaling pathway (103). On the other hand, Huyett et al., established a new 3D *in vitro* model for studying perineural invasion (PNI) in HNSCC by combining neurites generated from mouse’s dorsal root ganglion (DRG) and HNSCC cells in a semisolid matrix (107). Given that PNI is a potential risk factor in cSCC patients (108, 109), this model could potentially adapt to the study of PNI in cSCC. Recently, Sargenti and co-workers described the use of a new device called W8 (“weight”) (110), that allows a complete and accurate characterization of spheroids in terms of size, weight, and density using a fluidic-based measurement. This new tool holds promise of using spheroids in drug screening study (111), potentially applicable to cSCC.

Recent studies have demonstrated that the expression of PD-L1 (Programmed cell Death Ligand 1) in cSCC has a positive correlation with the risk of metastasis, which is in turn correlated with higher histological grade and tumor thickness (112, 113). It is known that PD-L1 binding to PD-1 on T cells prevents T cells from killing the PD-L1-containing cells, including the cancer cells. The immune checkpoint inhibitors bind to PD-L1 and block its binding to PD-1, leaving T cells free to kill cancer cells. In this regard, a novel 3D microfluidic platform that involves cSCC tumor-derived spheroids, freshly isolated from patient tumors, which contain not only tumor cells but also autologous tumor-infiltrating immune cells as well as stromal cells, will have a valid application in studies of the mechanisms of responsiveness and resistance to immunotherapy in patients with high risc cSCC, given the development of methods that provide a real-time response to the immune checkpoint inhibitors (114).

The latest development in 3D cell culture is represented by the organoids, which are composed of multiple cells type that self-organized in an organ-like structure (115). They can be originated from isolated tissue pluripotent stem cells (PSCs), including embryonic stem cells (ESCs) and induced pluripotent stem cells (iPSCs), grown in an appropriate microenvironment, such as solid matrix and specific exogenous signals (87, 116–118). Moreover, in contrast to spheroids, they have the ability of self-propagate and incorporate autologous lymphoid, myeloid, and other patient’s cell populations recapitulating the tumor contexts *in vivo* (119). The potential of organoids is referred to their ability in resembling the histology and genetics of the original organ *in vitro* (120), making them a useful tool for a wide range of applications in biomedical and cancer research. Genetic modifications of organoids allow studying the pathomechanisms of cancer, in a setting that recapitulate its physiological environment.

Additionally, the possibility to generate organoids from patient-derived tumoral cells opened new prospective for more efficient translational research and in the development of personalized treatments. The use of organoids derived from both healthy and tumor cells allows the screening of drugs that specifically targets tumor cells, avoiding toxic patient’s effects (121). Recently, a 3D culture system including organoids co-cultured with hematopoietic cells has been established. This system, in which tumor and immune cells co-exists, would be potentially useful in the study of immunotherapy responses (122–125).

Although organoids have been widely established from both primary and metastatic cancer biopsy (126–132), no work reported the use of this methods for cSCC research. However, skin organoids were efficiently generated starting from both ASCs (adult stem cells) (133) and PSCs (pluripotent stem cells) (134–136), indicating the potential effective application of this model for cSCC study by the use of tumoral keratinocytes.

#### Organotypic Culture

Mechanistic insights into tumor progression and validation of potential therapeutic targets can be achieved by human cSCC organotypic cultures. The skin reconstructs are a feasible solution to address the missing link between the conditions *in vivo* and the oversimplified 3D models *in vitro*. The organotypic cultures are able to mimic epidermis and dermis, including the cellular interactions of these layers together with metabolic and biomolecular properties, similar to *in vivo* condition. In recent years, 3D co-culturing methodologies of skin equivalent have evolved and reached important successes, so that skin reconstructs are widely used for pathophysiology studies. cSCC models, in fact, allow the analysis of the dynamics of tumor development, growth and invasive potential and offer a test system to evaluate new drugs (137), including the evaluation of photodynamic therapy (PDT) that is a well-established cSCC treatment.

*In vivo*, neoplastic cells, including the cSCC cells, reside in specific niches composed by stromal, immune, endothelial cells as well as connective tissue components, growth factors and cytokines, sustaining their status and modulating their activities. This tumor microenvironment actively collaborates with neoplastic cells and is crucial for cancer progression, controlling cancer cell differentiation, proliferation, invasion, and metastasis (138). For instance, the formation of an abnormal ECM that stimulates cancer progression starts with the activation of cancer associated fibroblasts (CAFs), which contribute to tissue fibrosis and matrix stiffness (139). In cSCC skin reconstructs, tumor cells, grow and actively interact with the different cell types and ECM, generating a tissue model with behavior similar to *in vivo* cSCC (140).

It is well-known that stem cell microenvironment is just as important for stem cell maintenance and functionality as stem cell intrinsic potential (141). Thus, the major achievement of advanced *in vitro* skin equivalents is their potential to recapitulate a microenvironment that is permissive for the establishment of a stem cell niche with long-term stem cell regeneration (142, 143). This advanced system has a great potential to understand the dynamic interactions between skin cells, cancer stem cells and their microenvironment. Interestingly, the long-term SCC skin reconstructs also recapitulate the respective degree of tumorigenicity of tumor-derived cells (64). In well-differentiated cSCC, laminin 322 and collagen IV are strongly expressed in the tumor nest near the basement membrane, while in poorly differentiated cSCC with higher potential to metastasize, these proteins are largely lost suggesting an enzymatic degradation of basement membrane (144). In cSCC organotypic cultures with cells in different stages of malignancy, the invasion phenotypes correlate with their individual degree of tissue organization, disorder in the basal membrane and the presence of matrix metalloproteinases (64).

Evidence has emerged about the decisive role of the stem cell niche also in carcinogenesis. Regulatory and inhibitory signals that are active in stem cell niche homeostasis may become disrupted during tumorigenesis. Khavari’s laboratory has shown that epithelial tissue in its native microenvironment can be surprisingly easy to convert to lethal malignancy, with only two genes needed to promote the formation of rapidly growing tumors resembling cSCC (145, 146). Ras retroviral transduction of primary human keratinocytes used to regenerate skin in immuno-deficient mice, seems to be sufficient to inhibit differentiation, to induce genes for angiogenesis and invasion, and only requires an additional genetic element, such as CDK4 overexpression, to facilitate escape from G1 growth restraints or nuclear factor-κB (NF-κB) blockade. However, this tumor does not metastasize when it originates from above the regenerated human epidermal basement membrane zone, location that mirrors its primary site of origin in human skin, instead of being injected directly into the bloodstream of the mice. Generation of human-tissue cancer models that contain intact human vascular and lymphatic elements are essential to study the mechanisms of cancer progression.

The complexity of the cSCC models used for research, commercial and clinical applications is largely dependent on the objectives. 3D culture methodologies can differ by scaffolds, matrices, and cell culture media, moreover the *in vitro* models can incorporate different patient-derived tissues/cells or engineered human cells. In the most common normal skin organotypic models, dermal equivalent consists of collagen type I gel embedded with dermal fibroblasts, de-epidermized dermis or self-assembled dermal sheets, and keratinocytes are seeded on top of this layer then exposed to air for epidermal stratification and keratinization (147).

In fresh cSCC biopsies cultured on fibroblast-seeded collagen matrices at the air-liquid interface, under serum-free condition, during 4-week culture period, invasive nests were seen in the direct vicinity of the original tumor biopsy, as well as in the dermal parts more distant from the original tumor biopsy (144). cSCC equivalents generated by seeding SCC-12B2 on dermal equivalents, containing normal human dermal fibroblasts, showed extensive invasion into the dermis-like compartment, while cSCC equivalent with cell line SCC13 showed disturbed attachment of the epidermal keratinocytes to the dermal equivalent. Therefore, considering the intrinsic characteristics of such cell lines, the models showed cell line-specific differences in basal membrane composition and related invasion.

To better mimic *in vivo* tumor microenvironment and to improve the structure and mechanical stability of ECM, Berning and collaborators found that a high fibroblast seeding density and growth supplementation together with the use of transwell technique, increased the thickness of the fibroblast-derived matrix-based dermal equivalent (64). This dermal equivalent represents self-assembled collagen fiber meshwork filled with a variety of human native proteoglycans and glycoproteins, which in its structural geometry largely resembles the papillary dermis of human skin. By seeding cSCC cells onto this matrix, this co-culturing system established a basal membrane excellent for monitoring tumor cell growth and invasion. Interestingly, when co-culturing the dermis equivalent with malignant tumorigenic HaCaT-RAS II-4, metastatic HaCaT-RAS A5-RT3, or cSCC-derived cell lines SCC12, SCC13, SCL-I, and SCL-II, the cSCC skin equivalents were generated that reflected the individual malignancy progression.

An additional cSCC skin equivalent model was generated with SCC13-derived rapidly adherent cells to collagen IV seeded onto the concave of the dermal reconstruct. This model was used to define the role of survivin in the context of squamous cell carcinoma-derived stem like cells, showing that survivin expression correlated with cSCC morphology and differentiation, promoting the malignant phenotype (46). After survivin silencing, cSCC skin equivalents displayed a reduced epidermal thickness, lower Ki-67 positive cell number, and decreased expression of MMP9 and psoriasin.

A new way to recreate cSCC model is to cultivate fibroblasts on a viscose fiber fabric for up to 14 days prior to tumor cultivation, allowing fibroblast to produce extracellular matrix and hence form solid tissue matrices (148). Subsequently, fresh cSCC tissue slices of approximately 3 mm thickness are carefully placed on top of the dermal equivalent and cultivated for up 21 days. In this model, cell heterogeneity and tumor microenvironment with CAFs, as well as vital immune cells are maintained, which are of particular interest for capturing current immunotherapy approaches. The model may serve as a platform to predict therapy response and to patient stratify as promising approach for precision medicine.

3D bioprinting is defined as a technology where the layer-by-layer deposition of bioinks is printed in a spatially defined manner as per a computer-aided designed structure to construct viable 3D structures. In the last decade, bioprinting methods have undergone remarkable advancements for studying cancer biology and screening anticancer agents (149, 150). 3D bioprinted skin models of cSCC tumors were bio-fabricated in 12-well transwell plates consisting of 3D-printed fibroblast embedded collagen-based dermis and an epidermal layer of normal keratinocytes with a basement membrane layer performed by Laminin/Entactin solution (151). After 1.5-h room temperature incubation, tissues were submerged and incubated at 37° for 4 days. A431 spheroids were pipetted onto the top surface of this tissue model, that were subsequently incubated for three more days submerged and 7 days with the air-liquid interface. Although cSCC was seeded as spheroids within the 3D bioprinted skin models, it exhibited vertical and radial growth. cSCC spheroids proliferated into and disrupted the normal epidermis. cSCC spheroids typically caused craters in the epidermis and extended into the dermis. Moreover, using RNAseq analysis, an increased expression of genes, such as S100A7, S100A8, S100A9, KRT6A, SERPINB3, SERPINB4, PI3, and RHCG, known to be upregulated in cSCC *in vivo*, was demonstrated. The model has been validated also at the functional level, through the investigation of pathology and confocal imaging, gene expression, and response to 5-Fluorouracil 48-h treatment (151).

Recent interest has focused on the generation of *in vitro* 3D vascularized skin models with dynamic perfusion and microfluidic devices known as skin-on-a-chip (152). Skin-on-chip is the best platform to study cell–cell interactions, to expose cells to drug candidates, mechanical strains, or even to study immune response (152). However, the currently used methods have to evolve as it is still not well established how to generate and maintain the cSCC skin-on-chip. Nonetheless, this is a powerful and very promising technology for personalized medicine approaches.

## *In vivo* Models

### Zebrafish Models

Zebrafish (*Danio rerio*), belonging to the Cyprinidae family, is a freshwater fish originating from South Asia, that has recently become very popular and effective as a model organism for developmental, toxicology and cancer research investigation. This is certainty due to its peculiarities, such as transparency and high reproductive capacity (153, 154). In fact, post-fertilization, zebrafish embryos rapidly develop ex-utero, and their larvae remain completely transparent, thus facilitating morphological observation of various structures and organ systems.

The exploration of a specific signaling pathway or drug effect is carried out using tissue-specific fluorescent reporter genes (155) or fluorescent labeled grafted cells (61). In fact, the creation of transparent albino strains, the so called *casper* (roy-/-, nacre-/-), that maintain transparency throughout their life, allow the live visualization of cancer cells progression immediately after the transplant and in adult animals (153, 156). Moreover, as compared to other animal models (e.g., mouse) the high fecundity of zebrafish contributes to a significant reduction in time requirements for each experiment, given the fertilization of 200–300 eggs per day every 5–7 days and that zebrafish have a longevity and generation time equivalent to those in mice (3–5 months).

The use of zebrafish for cancer research, and namely for skin cancer research, has been only recently introduced (154, 157). Treatments with chemicals for the development of random mutation in oncogenes have been shifted toward more biotechnological methods, with the development of transgenic animals enabling a dissection of a specific question in a temporal and spatial manner. The development of the transgenic zebrafish strains harboring p53 or BRAF or HRAS mutation has been notably central for melanoma related studies (158–160). Therefore, with this model, researchers have focused their attention on those factors that could be potentially responsible for tumor progression, for example, on the identification of step-by-step mutations or testing potentially effective drugs.

Other than transgenic animals, several studies consolidated the fact that the xenotransplantation of human cancer cell lines or primary cells represent a solid instrument to validate the function of specific signaling as potential therapeutic targets during tumor progression, through the evaluation of the grafted cell survival, proliferation, and metastasis. Moreover, patient-derived cells from blood malignancies or solid neoplasms further validate the relevance of such models in the context of a pre-clinical screening (161–163), with the aim to validate a patient specific drug response, and therefore providing a strong contribution to the development of a more personalized and precision medicine (164, 165). Furthermore, given that zebrafish adaptive immune system becomes mature at 2–3 weeks from fertilization (166), there is no need of producing immunodeficient strain for cell xenografting. On the other hand, the innate immune system is active starting from fertilization of the eggs, which means that it is potentially possible to observe the response produced by the grafting, thanks to the labeling of the zebrafish immune cells. In this direction, the use of the transgenic zebrafish line Tg (lysC:DsRed2) (167) or the Zebrafish mpeg1.1:GFP and mpeg1.1:mCherry reporters (155, 168) could be substantially used for the evaluation of the neutrophil or macrophage response to the grafting and contribute to the analysis of the inflammatory mediators produced by cancer cells.

In the context of squamous cancers, most of the research takes advantage of the xenografting procedure to evaluate the behavior of tumor cells in terms of proliferation and metastasis (169–174). As previously mentioned, patients with RDEB can develop a highly aggressive cSCC. Zebrafish model has been used to assess the role of Col7 (Collagen VII) in modulating cSCC-dependent angiogenesis (169), showing that the recombinant human Col7 is able to reverse the higher SCC angiogenesis in Col7 knock-down xenograft in transgenic fli1:EGFP (enhanced green fluorescent protein) zebrafish. The same strain has been used by Cichoń and co-worker to evaluate the function of the Axl tyrosine kinase receptor in cSCC (170). The expression of Axl correlates with poor prognosis and the induction of the EMT (epithelial to mesenchymal transition). The absence of Axl promoted a loss in tumor mass formation in zebrafish xenografting model, most likely by influencing the behavior of the cancer stem cells.

**FIGURE 3:**
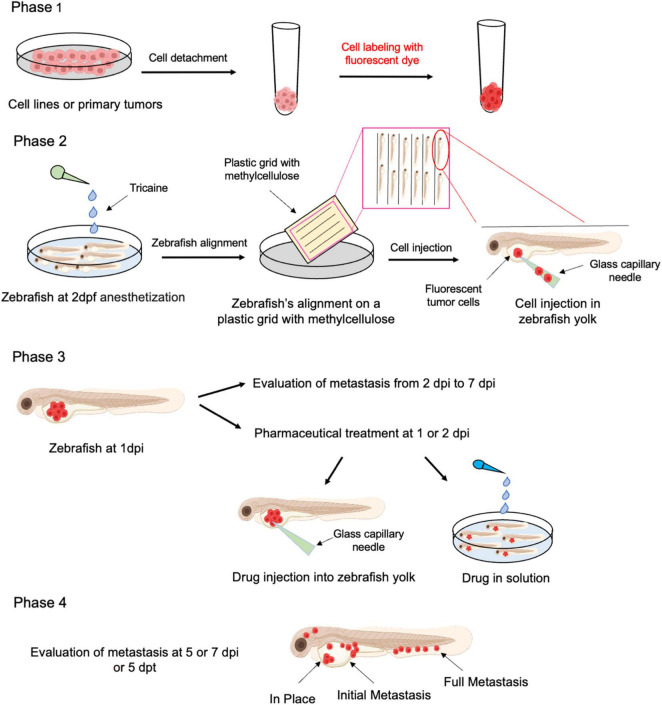
Schematic representation of xenotransplantation protocol in Zebrafish. Freshly isolated human cSCC cells or cSCC cell line grown as adherent cultures are harvested by trypsinization and labeled with a fluorescent live cell tracer **(Phase 1)**. Labeled cells (around 50 cells/fish) are injected into the yolk of 2 dpf zebrafish larvae, previously anesthetized **(Phase 2)**. Fish could be treated by drugs/compounds either by injection or addition to fishwater **(Phase 3)**. Follow up with metastasis and proliferation of cancer cell evaluation is performed up to 7 dpi **(Phase 4)**. Dpf, days post fertilization; dpi, days post injection; dpt, days post treatment.

Xenotransplant procedure and treatment are described in Figure 3. In the first phase, tumoral cells, cultured in 2D condition for expansions (e.g., cell lines, primary tumor cells, gene × silenced or overexpressed cells, etc.), are detached with trypsin and stained with a fluorescent dye. In this procedure, a lipophilic tracer is usually employed that labels the lipophilic membrane of viable cells. Secondly, dechorionated embryos at 2 days post fertilization (dpf) are anesthetized with tricaine, placed on a plastic grid immersed in a solution of methylcellulose and aligned along the grid-lanes. Labeled tumor cells are then loaded in a glass capillary needle and microinjected into the zebrafish yolk (about 50 cells/embryo), using a WPI PicoPump apparatus. In the third phase, at 1-day post injection (dpi), those embryos displaying cells in the circular vessels are discarded. Subsequently, the metastatic process is evaluated every day from 2 to 7 dpi. On the other hand, embryos could be treated with a pharmaceutical or natural drug, by injecting and/or dissolving it into the fish water, to evaluate its effect on tumor proliferation and invasion *in vivo* in real time. Finally, xenografted embryos are grown at 33°C, monitored daily and documented from 2 dpi or 2 days post treatment (2 dpt) up to 7 dpi or 4 dpt. At the experimental endpoint, metastases are quantified and classified as follows: (i) in place, tumor cells confined to the site of injection; (ii) initial metastases, tumor cells spread from yolk to near organs (classically heart, swim bladder and pharynx); (iii) full metastases, tumor cells in distant organs, such as brain, skeletal muscle, and trunk. Moreover, the fluorescence intensities emitted from injected tumor cells could be quantified by imaging software (e.g., ImageJ) to obtain an estimated of the number of live tumoral cells (61, 164).

### Mouse Models of Cutaneous Squamous Cell Carcinoma

Preclinical mouse models of cSCC are important and widely used research tools in the field of experimental squamous skin carcinogenesis. These *in vivo* models have provided significant insights into the cSCC pathogenesis. Here we discuss several types of commonly used mouse cSCC models, including: (1) cell line-derived (xeno- and syngeneic) transplantation models, (2) autochthonous environmental carcinogen-induced models, including chemically-induced and ultraviolet radiation-induced mouse cSCC models, and (3) genetically engineered mouse (GEM) models of cSCC.

#### Cell Line-Derived Xenograft Transplantation Models of Cutaneous Squamous Cell Carcinoma

The cell line-derived xenograft models are among the most commonly used *in vivo* models in basic and translational cancer research (175, 176). These models have contributed greatly to advancing our understanding of the mechanisms of cancer development and enabling identification and validation of potential cancer regulatory genes and pathways, as well as preclinical candidate drug testing. However, relatively few human cSCC cell lines have been developed up to now (Table 1). Among them, SCC13 and A-431, are widely utilized in mouse xenograft models for preclinical squamous cell carcinogenesis research (177–190). In addition, the panel of the cSCC cell lined established at the University of Turku includes five primary (UT-SCC-12A, UB-SCC-91, UT-SCC-105, UT-SCC-111, UN-SCC-118) and three metastatic (UT-SCC-7, UT-SCC-59A, UT-SCC-115) cSCC cell lines (191). A number of published studies employed the metastatic UT-SCC-7 cell line in mouse cSCC tumor xenograft experiments (192–195). Furthermore, several publications reported using primary cSCC cell lines SRB-1 and SRB-12 (196), and metastatic cSCC cell line COLO16 (197) in mouse cSCC tumor xenograft models (198–200). More recently, a large panel of sixteen comprehensively characterized patient-derived primary and metastatic cSCC cell lines have been developed in Leigh’s laboratory (201). The *in vivo* tumorigenicity of the majority of these cell lines (14 of the 16 cell lines) have been studied in mouse cSCC tumor xenograft models. The examination of the growth kinetics of xenograft tumors derived from the different cSCC cell lines included in this panel provided valuable information about the suitability of the specific xenograft models for the short-term or the long-term drug testing. These cell lines provide a useful resource for preclinical cSCC research, and many of them have already been utilized in numerous collaborative studies as reviewed in Hassan et al. (201).

**FIGURE 4:**
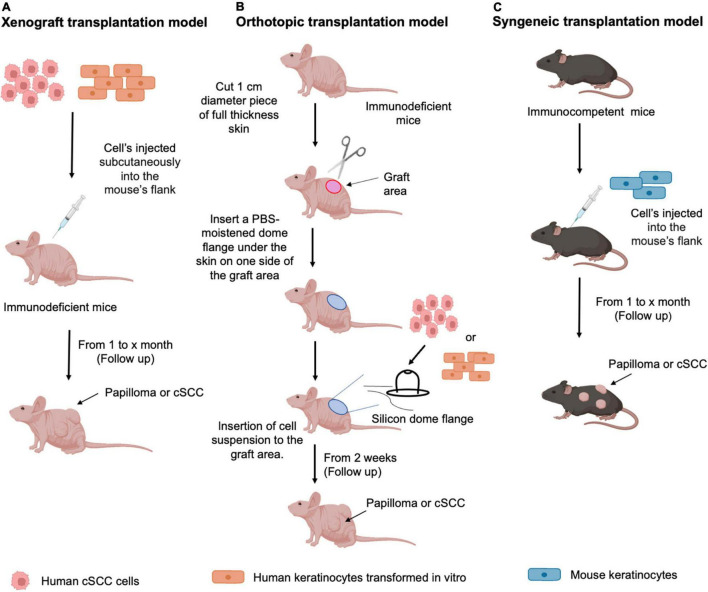
Transplantation mouse models of cSCC. Schematic representation of the protocols used for **(A)** Xenograft transplantation model: human cSCC cells or human keratinocytes transformed *in vitro* are subcutaneously inoculated into the appropriate anatomic site (flank) of an immunodeficient murine host. After cell injection, follow up is performed starting from 1 month to evaluate papilloma or cSCC tumor formation; **(B)** Orthotopic transplantation model: Immunodeficient murine host is prepared for the grafting procedure by cutting 1 cm of full-thickness skin to allow the insertion of the silicone dome flange. Human cSCC cells or human keratinocytes transformed *in vitro*, mixed with mesenchymal cells, are inserted into the dome. After cell injection, follow up is performed starting from 2 weeks to evaluate papilloma or cSCC tumor formation **(C)** Syngeneic transplantation model: murine keratinocytes harboring specific characteristics, as described in the text, are injected into the mouse’s flank. After cell injection, follow up is performed starting from 1 month to evaluate papilloma or cSCC tumor formation.

To ensure successful engraftment and prevent immune rejection, human cSCC cells are typically inoculated into immunodeficient murine hosts, such as the T-cell-deficient athymic Nude (nu/nu) mice, the T- and B cell–deficient severe combined immune deficiency (SCID), or the extremely immunocompromised NSG [NOD/SCID/IL2 receptor gamma chain (IL2Rγ)-null] and NOG (NOD/Shi-SCID/IL2Rγ-null) mouse strains, which lack mature T cells, B cells, and NK cells, and have dysfunctional macrophages and dendritic cells (176). Notably, the immunodeficient background of murine hosts precludes using mouse xenograft models for study of host immune cell function in tumor development or evaluation of immune-modulating interventions (176, 202). Moreover, given that human cSCC cell line-derived xenograft tumors are largely generated in an ectopic manner, that is by subcutaneous injection of tumor cells into a convenient anatomical location, such as flank (Figure 4A), the microenvironment of the xenograft cSCC tumors does not closely mimic that of their tumors of origin. In this regard, the orthotopic cell transplantation into the skin as the organ of origin (Figure 4B) would more faithfully simulate human tumor cSCC microenvironment (203). However, orthotopic transplantation models are technically demanding and relatively seldom used for studies involving the established human cell-line-derived xenografts (176, 202). Of note, a model of human cSCC tumorigenesis in a murine xenograft model, involving co-expression of oncogenic HRAS and either cyclin-dependent kinase 4 (CDK4) or the nuclear factor-κB inhibitor IκBα in human keratinocytes, has also been described (154, 155).

Among other major limitations of the cell-derived xenograft models are potential irreversible alterations in gene expression due to a long-term *in vitro* culture as well as loss of genetic heterogeneity (175). Patient-derived xenograft (PDX) models, which are generated by subcutaneous implantation of fresh surgically obtained samples of patient tumors into immunodeficient mice, appear to more faithfully preserve genetic and histologic characteristics of their tumors of origin, compared with cell-derived xenograft models, and therefore serve as a promising tool for predicting therapy response and for personalized medicine (175, 176). For example, a recent study employed patient cSCC-derived xenografts to investigate the mechanisms of resistance to EGFR-targeted therapy and reported that the resistance can be overcome by a combined treatment with EGFR and FGFR inhibitors (204). However, the use of PDX models in the studies of cSCC is relatively uncommon (175).

Despite the above-mentioned considerable shortcomings of the cell line-derived xenograft transplantation model, this approach, combined with *in vitro*, transcriptomic, genomic, proteomic, metabolomic, and bioinformatic methodologies, continues to contribute novel insights into the mechanisms of squamous cell carcinogenesis. Based on the discovery of a critical transcriptional feed-forward circuit consisting of *PITX1*, *SOX2*, and *TRP63* that promoted self-renewal of basal tumor-propagating cSCC cells and cSCC growth while repressing *KLF4*-dependent differentiation, Sastre-Perona and colleagues proposed a possibility of developing differentiation-inducing therapeutic strategies for cSCC (182). In addition, targeting cSCC cancer stemness by suppressing the long non-coding RNA (lncRNA) HOTAIR and its downstream effectors Sp1 and DNMT1 have been suggested to benefit cSCC treatment (190). Moreover, protein translation initiation, as well as enhanced glycolytic metabolism have also recently emerged as novel potential therapeutic targets for cSCC (181, 185). Of note, several tumor-derived complement system components have been shown to promote cSCC progression *in vivo* and implicated as biomarkers and potential cSCC intervention targets (192–195, 205). A superior efficacy of a combination treatment with chemotherapeutic drug cisplatin and diet-derived natural cancer-preventive agent sulforaphane (SFN) was demonstrated in an *in vivo* cSCC xenograft model, compared to treatment with each agent alone, suggesting that cisplatin/SFN combination treatment may be a useful therapy for aggressive cSCC (177). Additional studies have revealed several important SFN cancer-preventive molecular targets, including YAP/ΔNp63α signaling axis, that is inhibited by SFN to suppress cSCC tumor growth (180), and type 2 transglutaminase (TG2), the enzymatic activity of which is suppressed by covalent and irreversible binding of SFN to TG2 to inhibit the TG2-medicated maintenance of aggressive cSCC cancer phenotype (206). Notably, using orthotopic model of cSCC, Katarkar and colleagues have shown that genetic silencing or pharmacological inhibition of NOTCH1 in CAFs suppressed orthotopic cSCC tumor growth, suggesting that NOTCH1 in the CAF compartment of the tumor stroma is a potentially useful target for stroma-centered anti-cSCC therapy (186). Future studies are needed to validate the translational potential of these exciting pre-clinical discoveries.

#### Syngeneic Transplantation Models of Cutaneous Squamous Cell Carcinoma

There is presently a relative scarcity of syngeneic transplantable cSCC tumor models in immunocompetent mice (Figure 4C), which is a major hurdle in studying the cSCC tumor immune microenvironment and the mechanisms of immunotherapy resistance.

Pam 212 cell line was established from neonatal BALB/c mouse keratinocytes that underwent a spontaneous malignant transformation *in vitro*; it produced well-differentiated but rapidly growing SCC in BALB/c syngeneic hosts (207). In addition, metastatic variant cell lines were derived following *in vivo* tumor progression of the parental Pam 212 cell line, providing a model to study cSCC metastatic progression in immunocompetent BALB/c hosts (208).

Squamous cell line PDV was derived from *in vitro* cultured neonatal C57BL/6 mouse keratinocytes treated with carcinogen 9,10-dimethyl-1,2-benzanthracene (DMBA), and harbors an activating codon 61 mutation in the *Hras* oncogene (209). Intradermal injection of PDV cells into flanks of syngeneic C57BL/6 hosts led to development of well-differentiated cSCC tumors only in about 10–20% of injection sites, attesting to the immunogenicity of these tumors in syngeneic immunocompetent hosts (209, 210). PDV tumor rejection was demonstrated to be dependent on the presence of T-cells (210–212).

PDVC57 cell line was derived from one of PDV tumors established following intradermal injection of PDV cells into a syngeneic C57BL/6 host and represents *in vivo* clonal expansion of the parental PDV cancer cell in mouse skin (209). Compared with the PDV cell line, PDVC57 cells were more tumorigenic in syngeneic C57BL/6 immunocompetent hosts (209, 213). More aggressive growth of PDVC57 tumors in syngeneic hosts was associated with increased intra-tumoral infiltration of tumor-associated macrophages (TAMs), as well as decreased infiltration of dendritic and T-cells; moreover, PDVC57 tumors had higher activity of TAM-derived arginase, an enzyme implicated in promoting tumor growth (213). Notably, topical arginase inhibition reduced PDVC57 tumor growth in immunocompetent C57BL/6, but not in immunodeficient *Rag1*-null mice which lack mature T- and B-cells. In addition, the combination of topical arginase inhibitor and systemic anti-PD-1 immunotherapy with nivolumab enhanced ant-PD-1 efficacy, led to synergistic decrease in tumor growth, and promoted anti-tumor immunity (213). These findings support the potential benefit of topical arginase inhibition as a therapeutic strategy for cSCC, as well as highlight the utility of implantable syngeneic mouse models as valuable tool for investigating the contribution of host immune cells to the development of cSCC tumors. Another approach to model *in vivo* anti-cancer immune responses would involve establishing and characterizing murine cSCC cell lines derived from UVR-induced mouse skin SCC in diverse mouse strains, followed by implantation of these cell lines into the matching syngeneic hosts with intact immune system.

#### Carcinogen-Induced Models of Cutaneous Squamous Cell Carcinoma

##### Murine Chemically Induced Skin Carcinogenesis Model

**FIGURE 5:**
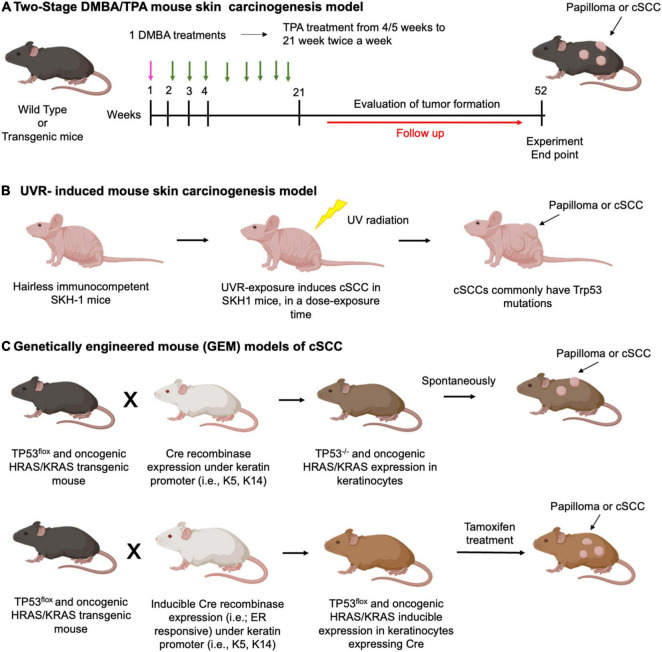
Carcinogen-induced models of cSCC. Schematic representation of the protocols used for **(A)** Two-stage DMBA/TPA mouse skin carcinogenesis model. DMBA treatment (pink arrows), TPA treatment (green arrows). Typically, a single DMBA treatment (week 1) is followed by sustained TPA treatments up to 20 weeks. From 21 to 52 weeks a follow up of the animals will evaluate tumor formation; **(B)** UVR-induced mouse skin carcinogenesis model: hairless immunocompetent SKH-1 mice undergo UVR exposure, which typically induces Trp53 mutation. cSCC formation depends on dose and time of exposure. **(C)** Example of genetically engineered mouse (GEM) models of cSCC. Upper panel: p53 Flox mice and oncogenic HRAS or KRAS expressing mice are mated with transgenic conditional keratinocyte Cre recombinase expressing mice. Cre expression is constitutive under control of a keratin promoter. The offspring with loss of p53 or oncogenic HRAS and KRAS expression spontaneously develop papilloma or cSCC.; lower panel: mating scheme and tumor formation works as described for the upper panel. However, Cre protein is fused with the estrogen receptor (ER), generating a CreERT protein that works only after tamoxifen treatment.

In the well-established murine two-stage 9,10-dimethyl-1,2-benzanthracene (DMBA)/12-*O-*tetradecanoyl phorbol-13-acetate (TPA)- induced skin squamous carcinogenesis model, mice are typically treated with a single subcarcinogenic dose of an initiating agent, mutagenic carcinogen DMBA, applied topically to mouse skin, followed by a long-term twice weekly topical application of the non-mutagenic, non-carcinogenic tumor promoter, irritant inflammatory agent TPA (Figure 5A). As a result of this treatment regimen, within 10–20 weeks of promotion, mice develop premalignant squamous tumors papillomas harboring activating mutations in the *Hras* oncogene, followed by a progression of a portion of papillomas to cSCC within 20–50 weeks (214, 215). Notably, DMBA-induced mouse skins SCCs have been demonstrated to share a number of commonly mutated genes with human SCC, supporting the pre-clinical relevance of this mouse model (216).

The two-stage DMBA/TPA chemical skin carcinogenesis model has been instrumental in providing important insights into the mechanisms of squamous cancer pathogenesis, including the emergence of the important concepts of tumor initiation, promotion, premalignant progression, and malignant conversion [reviewed in (217)]. It is well-established that initiating oncogenic mutations persist over time, suggesting that these mutations occur in long-lived epidermal stem cells. The identity and the location of the initiated cell determines the probability of malignant growth (218). Both hair follicle and interfollicular epidermal stem cell function as cells of origin of squamous tumors in mouse skin [reviewed in (28)]. Of note, in addition to activating *Hras* mutations, DMBA-initiated epidermal cells contain a multitude of other mutations, including other known cSCC driver mutations (214). Importantly, evidence from the two-stage mouse skin carcinogenesis model suggests that these mutations are insufficient for skin tumor development in the absence of the promotion phase; in fact, the promotion phase has been suggested to be the rate-limiting phase in the tumor formation, underscoring the crucial role of non-tumorigenic promoting agents, environmental or endogenous, in squamous cancer pathogenesis, both in mouse models and in humans [reviewed in (214)]. In the two-stage model, tumor promoter TPA stimulates the clonal growth of initiated cells by activating signaling pathways linked to proliferation, as well as induces chronic tissue inflammation. The malignant conversion of papillomas to cSCCs is associated with progressive chromosomal abnormalities and loss of function mutations in p53 tumor suppressor gene. Susceptibility to two-stage mouse skin carcinogenesis is dependent on mouse genetic background and the doses of the initiating and promoting agents (215).

The biological relevance of the two-stage mouse skin carcinogenesis model to human cancer, including its salient features such as *de novo* tumor formation, the multistage pathogenesis of cancer, and the ability to replicate phenotypic and genetic heterogeneity of human tumors, as well as the utility of this model to yield powerful insights into the mechanisms of squamous cancer development have been well-recognized (175, 217). On the other hand, long latency and variable penetrance are among the major limitations of this model, as well as of other environmental carcinogen-induced models, including UVR-induced murine cSCC model (discussed below), contributing to a relatively infrequent use of these models in current cancer research (175).

Nonetheless, the two-stage DMBA/TPA mouse skin carcinogenesis model continues to yield exciting new discoveries in the field. Thus, Alimirah and colleagues have recently reported that senescent cells act as tumor promoters, rather than tumor initiators, and stimulate skin carcinogenesis through activation of p38 MAPK and MAPK/ERK signaling (44). These findings support the potential benefit of targeting chemotherapy-induced senescent cells as a strategy to reduce occurrence of second cancer in the future. A recent study demonstrated that RNA-binding protein tristetraprolin that binds to AU-rich elements (AREs) in the 3′-untranslated regions of mRNAs and targets ARE-containing mRNAs for degradation, is an important regulator of skin carcinogenesis and may represent a useful therapeutic target (219). Of note, the critical role of the interleukin (IL)-33/regulatory T cell (Treg) axis in chronic inflammation-induced tumor-promoting environment both in the skin and colon has been recently elucidated, suggesting a promising therapeutic strategy for the treatment and prevention of cancers associated with chronic inflammation (220). Strickley and colleagues have reported that immunity to commensal papillomaviruses was protective against skin carcinogenesis induced by DMBA/TPA or by UVR in a manner dependent on CD8^+^ T cells (221). These findings revealed a previously unappreciated beneficial role for commensal viruses and supported the utility of immune-based approaches, such as enhancing anti-HPV immunity, to suppress skin cancer development and improve the efficacy of immune checkpoint inhibitor immunotherapy against SCC. Furthermore, micro-RNA *mi-R22* was found to activate Wnt/β-catenin signaling in cancer stem cells (CSCs) to promote tumor initiation, progression and metastasis of DMBA/TPA-induced mouse skin SCC (222). Genetic depletion of a homeobox transcription factor Dlx3 in skin epithelium was reported to promote DMBA/TPA-induced mouse skin carcinogenesis through activation of the EGFR–ERBB2 signaling pathway, underscoring the potential benefit of targeting this pathway in cSCC (39). Of note, the DMBA/TPA model, together with *in vitro* mechanistic studies and the results of the analysis of human tumors, was instrumental in uncovering the novel role of thyroid hormone in promoting progression and invasiveness of cSCC *via* the induction of EMT, suggesting that pharmacological inhibition of thyroid hormone signaling may suppress metastatic capacity of cSCC (223).

##### Ultraviolet Radiation-Induced Mouse Cutaneous Squamous Cell Carcinoma Model

The UVR from the sunlight exposure is a major environmental carcinogen that has been implicated in the development of keratinocyte cancers including both basal cell carcinoma (BCC) and cSCC. The solar UVR spectrum is composed of 95% UVA (320–340 nm) and 5% UVB (290–320 nm) (224). UVB and UVA components penetrate the epidermis and the underlying dermis, respectively, and induce different forms of DNA damage, as well as extensive deleterious changes in the skin microenvironment which contribute to immunosuppression, photoaging and photocarcinogenesis (224). An outbred (uncharacterized/non-pedigreed) immunocompetent SKH-1 hairless mouse strain is commonly used in studies of UVR-driven mouse skin carcinogenesis as a highly relevant model for UVR-induced human cSCC (225) (Figure 5B). To accurately model human cSCC, Knatko and colleagues have chronically (twice a week for 15 weeks) exposed SKH-1 hairless mice to solar-simulated UVR, which resulted in the development of mouse cSCC that shared similar histopathology with human cSCC (226). Furthermore, whole exome sequencing of genomic DNA isolated from tumor tissue revealed a significant similarity between mouse cSCC that develop in this model and human cSCC tumors in terms of the mutational landscape and UVR-induced mutational signature. These findings thus validate a high biological relevance of this mouse skin carcinogenesis model to human disease and highlight its utility for mechanistic studies and for assessing prospective prevention and treatment approaches (226). Zhang and colleagues used solar-simulated UVR exposure of SKH-1 mice to model the progression from premalignant AK to cSCC and reported a critical importance of the loss of retinoic acid receptor-related receptor alpha (Rorα) in promoting this progression (89).

### Genetically Engineered Mouse Models of Cutaneous Squamous Cell Carcinoma

Genetically engineered mouse models (GEMMs) usually allow for much higher (in many cases 100%) tumor prevalence and incidence than models employing chemical or UVR-induced skin carcinogenesis. Due to the homogenous nature of cancer driver or tumor suppressor mutations, GEMMs provide a powerful platform to gain mechanistic insights regarding tumorigenesis and metastasis, and to study drug responsiveness and resistance (175, 176). The important advances in genetic engineering technology, which enabled the generation of diverse GEMMs, as well as various strategies for gene targeting, such as germline or cell type-specific overexpression or knockout of single or multiple genes of interest in constitutive or inducible manners, were recently reviewed (175, 176). In addition, the strategies for expressing oncogenes and/or deleting tumor suppressor genes to stimulate tumor formation in the different epidermal cell lineages have been recently discussed (28) (Figure 5C). With the development of novel genetic manipulation methods, such as clustered regularly interspaced short palindromic repeats (CRISPR)/Cas9 gene editing technology (227), multiple genes can be rapidly targeted to focus on the interactions of specifically selected genes. Importantly, as GEMMs exhibit *de novo* tumor development in an immunocompetent hosts, these models can be utilized for studying cancer-preventive or immune-modulatory interventions (175). In addition, GEMMs can be combined with chemical or UVR carcinogenesis studies or with PDX to simultaneously harness the advantages of multiple approaches.

Genetically engineered mouse models combined with two-stage chemical mouse skin carcinogenesis are ideal tools to study the roles of different genes and signaling pathways in control of distinct stages of tumor development and progression (215). Mouse models of squamous tumor development resulting from a combination of overexpression of activated *Kras^G12D^* oncogene and loss of function of the *p53* tumor suppressor showed that the identity of the cell of origin contribute to the diversity of the resulting tumor types (28). Thus, squamous tumors derived from the interfollicular epidermis were found to be less aggressive, while those derived from hair follicle stem cells showed increased tendency of EMT and metastatic potential (28, 228).

Genetically engineered mouse models are essential in confirming the oncogenic role of various mutations and their place in the complex proliferative or inhibitory signaling pathways in cancer, thus identifying new potential targets of therapeutic interventions. Studies using transgenic mouse models showed that YAP and TAZ, important downstream regulators of the Hippo pathway which control cell proliferation and differentiation, are essential for both basal and squamous cell carcinoma initiation (229), and that YAP drives cSCC formation and progression (229). Of note, wound healing response was shown to synergize with YAP and drive cSCC progression to an aggressive spindle cell carcinoma (spSCC) subtype (229).

In skin tumors, cSCCs can arise from basal cell carcinomas as a result of basal to squamous cell carcinoma transition (BST), and this tumor plasticity poses a significant drug resistance problem. A mouse experimental model of BST, in conjunction with multi-omics approach, was employed to identify c-Fos transcription factor as a central player in regulating the processes of tumor plasticity (230).

TAp63, a potent tumor and metastasis suppressor, is a member of the p53 family. TAp63-null mice were reported to have an increased susceptibility to UVR-induced cSCC tumorigenesis; it was further shown that TAp63-regulated miRNAs suppressed cSCC development through the inhibition of a network of cell-cycle regulating genes (231).

The role of ERK5 MAPK in the inflammatory cell compartment of the tumor stroma during squamous carcinogenesis was studied using GEMMs. These studies showed that myeloid ERK5 deficiency suppressed tumor growth by blocking protumor macrophage polarization *via* inhibiting STAT3 activation (232) and that ERK5-dependent p21 expression promoted macrophage proliferation associated with tumor growth and metastasis (233). These findings support the potential benefit of targeting ERK5 in the macrophage compartment of the tumor stroma and underscore the utility of using the GEMMs for preclinical testing of the potential therapies targeting the tumor microenvironment.

## Conclusion

In summary, cSCC studies benefit from the constantly increasing level of biomolecular technologies and the huge amount of information continually derived from basic and translational research. Both *in vitro* and *in vivo* models are important tools to study cSCC pathogenesis. As these pre-clinical models continue to be refined, their clinical relevance will undoubtedly continue to improve, leading to better translatability and novel opportunities for therapeutic interventions.

## Author Contributions

All authors contributed to writing and revising the manuscript and gave final approval.

## Conflict of Interest

The authors declare that the research was conducted in the absence of any commercial or financial relationships that could be construed as a potential conflict of interest.

## Publisher’s Note

All claims expressed in this article are solely those of the authors and do not necessarily represent those of their affiliated organizations, or those of the publisher, the editors and the reviewers. Any product that may be evaluated in this article, or claim that may be made by its manufacturer, is not guaranteed or endorsed by the publisher.
